# Etiologies of community-acquired febrile illness identified by TaqMan Array Card qPCR on blood samples: a systematic review and meta-analysis

**DOI:** 10.1128/jcm.00101-26

**Published:** 2026-02-27

**Authors:** Carl Boodman, Nitin Gupta, Cansu Cimen, Johan van Griensven, Matthew P. Cheng, Cedric P. Yansouni, Emmanuel Bottieau

**Affiliations:** 1 Division of Infectious Diseases, Department of Internal Medicine, University of Manitoba 8664, Winnipeg, Manitoba, Canada; 2 Department of Clinical Sciences, Institute of Tropical Medicine 37463 , Antwerp, Belgium,; 3 Department of Medical Sciences, University of Antwerp 37463, Antwerp, Belgium; 4 Department of Infectious Disease, Kasturba Medical College, Manipal Academy of Higher Education 76793, Manipal, India; 5 Department of General Internal Medicine, Infectious Diseases and Tropical Medicine, Antwerp University Hospital 60202, Antwerp, Belgium; 6 Divisions of Infectious Diseases and Medical Microbiology, McGill University Health Centre 54473, Montréal, Québec, Canada; 7 JD MacLean Centre for Tropical and Geographic Medicine, McGill University 54473, Montreal, Québec, Canada; Maine Medical Center Department of Medicine, Portland, Maine, USA

**Keywords:** community-acquired infection, epidemiology, fever, PCR

## Abstract

Community-acquired febrile illness presents a significant diagnostic challenge. The TaqMan Array Card (TAC) enables simultaneous detection of many bacterial, viral, parasitic, and fungal pathogens. This systematic review aimed to characterize the etiologies of community-acquired fever diagnosed by TAC directly on blood specimens. A systematic search of PubMed, Scopus, Embase, and Web of Science (from inception to January 2026 in English) identified studies using TAC to detect pathogens directly in human blood to identify the etiology of community-acquired febrile illness. Eligible studies were appraised using a modified Joanna Briggs Institute (JBI) checklist. Pooled proportions and meta-analyses were conducted using a random-effects model. A total of 16 studies comprising 8,937 participants from 12 African and Asian countries met inclusion criteria. TAC detected at least 1 pathogen in 0.43 (95% CI [0.32; 0.54]), with high between-study heterogeneity (*I²* = 99%). Parasitic infections were most common (56%), nearly all due to *Plasmodium* spp., followed by bacterial (26%), viral (22%), and fungal (0.5%) etiologies. *Plasmodium* spp., *Rickettsia* spp., dengue virus, Lassa fever virus, *Streptococcus pneumoniae, Orientia tsutsugamushi*, *Mycobacterium tuberculosis* complex, and *Salmonella* spp. were the most frequently identified pathogens. Culture-negative bacteria accounted for 56% of bacterial detections. Positivity with more than 1 organism occurred in 6% of cases. TAC showed strong concordance with blood culture for culturable organisms (positive agreement: 73%, 95%CI [0.53; 0.89]; negative: 98%, 95%CI [0.95; 1.00]). TAC provides culture-independent pathogen detection and reveals overlooked causes of febrile illness, such as culture-negative bacteria. Its application may improve clinical diagnosis and surveillance programs.

**IMPORTANCE:**

Accurate identification of the causes of community-acquired febrile illness is essential for guiding clinical decision-making, surveillance programs, and public health response. This systematic review and meta-analysis synthesizes data from more than 8,937 patients across Africa and Asia tested with blood-based TaqMan Array Card (TAC) assays. TAC enabled broad, culture-independent detection of bacterial, viral, parasitic, and fungal pathogens—revealing both expected causes, such as *Plasmodium* spp. and dengue virus, and neglected culture-negative bacteria including *Orientia tsutsugamushi*, *Coxiella burnetii*, and *Rickettsia* spp. By uncovering these underrecognized etiologies, TAC provides valuable pathogen-level data to strengthen fever surveillance, inform outbreak detection, and refine regional disease prioritization frameworks. These findings highlight the potential of high-throughput molecular diagnostics to enhance infectious disease control, particularly in resource-limited settings.

## INTRODUCTION

Community-acquired febrile illness remains a clinical and public health challenge, with causes like malaria, dengue virus infection, enteric fever, and leptospirosis presenting with overlapping symptoms (1, 2). In low-resource settings, the challenge of identifying the etiology of undifferentiated febrile illness is exacerbated by limited access to diagnostic infrastructure and resources (3). Among 14 surveyed Sub-Saharan African countries, only 1% of the laboratories could perform clinical bacteriology testing, a core modality for identifying the etiology of undifferentiated febrile illness (4). This finding underscores the inadequate characterization of the causes of community-acquired fever, which hampers effective clinical decision-making, surveillance initiatives, and antimicrobial stewardship efforts (3). While a broad spectrum of bacterial, viral, fungal, and parasitic pathogens are known to cause community-acquired febrile illness (and produce infections detectable in blood), most studies investigate only a limited subset of potential etiologies (5).

The TaqMan Array Card assay (TAC) is a high-throughput, microfluidic 384-well parallel monoplex quantitative polymerase chain reaction (PCR) platform that enables the simultaneous detection of 40–50 pathogen-specific genetic targets in a single reaction (6). The assay facilitates the identification of a wide array of bacterial, viral, fungal, and parasitic pathogens, including DNA and RNA organisms (7). Each card is preloaded with lyophilized primers and probes, with a closed-system structure that reduces the risk of contamination (6, 7).

Although several multiplex PCR platforms are available for identifying the etiology of febrile illness from blood specimens, in many cases, their design, intended users, and target use cases yield panels that omit key pathogens. For example, the BioFire Global Fever Panel (BioFire Defense) detects 19 pathogens, including viral hemorrhagic fever (VHF) agents and other epidemic threats, but excludes common bacterial causes of bloodstream infection such as *Streptococcus pneumoniae* and *Salmonella* spp. (8). The SeptiFast assay (Roche Diagnostics, Mannheim, Germany) was developed to diagnose etiologies of sepsis in high-income settings, omitting viruses, parasites, and vector-borne agents entirely (9). Both platforms lack the ability to detect several bacterial pathogens that often yield negative culture results, such as *Rickettsia* species, *Orientia tsutsugamushi*, *Coxiella burnetii*, and *Bartonella* species. Recent studies suggest these pathogens may be more common causes of bloodstream infection than previously recognized (10, 11). In South India, scrub typhus (*Orientia tsutsugamushi*) incidence was 6.0 cases per 1,000 person-years, placing it among the most common infections in the region (10). In Tanzania, culture-negative bacterial zoonoses were common causes of hospital admission for febrile illness, with diagnoses including leptospirosis, Q fever, spotted fever group rickettsioses, and typhus group rickettsioses (12). Because TAC uses a modular format, validated primers and probes for relevant pathogens, such as culture-negative bacteria, can be readily incorporated, an advantage over fixed-content multiplex platforms.

Among adults and/or children with community-acquired febrile illness of any duration, what is the positivity rate and etiologic spectrum of TAC performed directly on blood samples?

We conducted a systematic review and meta-analysis of studies using blood-based TAC in patients with community-acquired, undifferentiated febrile syndromes to estimate pooled TAC positivity; characterize the distribution of bacterial, viral, parasitic, and fungal detections (including co-infections); and evaluate agreement relative to blood culture for culturable organisms.

## MATERIALS AND METHODS

### Systematic literature search strategy

**Fig 1:**
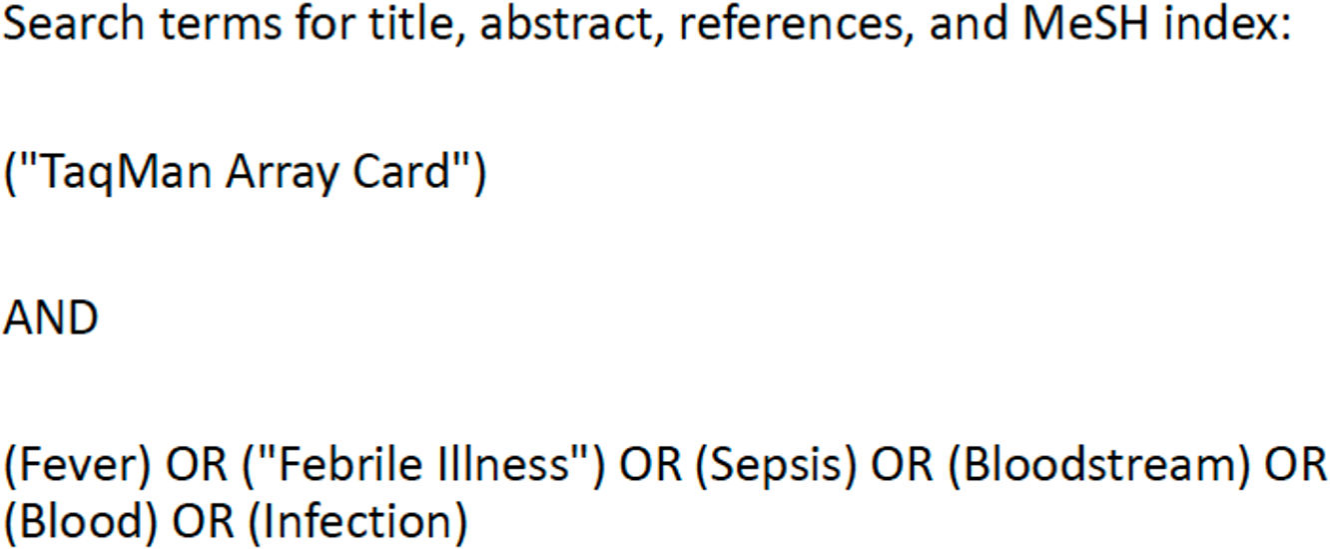
Search terms applied to the databases and registries to identify relevant publications.

We searched databases in PubMed Central/MEDLINE, Scopus, Embase, and Web of Science and the trial registers www.ClinicalTrials.gov and https://trialsearch.who.int/Default.aspx from database inception to 17 June 2025 to identify publications containing specific search terms (Fig. 1). We searched for titles and abstracts using the following search string: {(“TaqMan Array Card”) AND (Fever OR “Febrile Illness” OR Sepsis OR Bloodstream OR Blood OR Infection)} (Fig. 1). An additional search was conducted to identify articles published between 17 June 2025 and 19 January 2026 to ensure that the review remained up to date. We searched reference lists of selected publications to identify other reports. While search terms were run in English, no language or geographic restrictions were placed. This review followed the Preferred Reporting Items for Systematic Reviews and Meta-Analyzes (PRISMA) guidelines for systematic literature reviews and was registered in the International Prospective Register of Systematic Reviews (PROSPERO; identifier CRD420251046495) (13, 14).

### Study selection

We evaluated any publication containing infectious disease diagnoses made by TAC performed directly on human blood specimens to identify the etiology of community-acquired febrile illness of any duration among adults and children (other than neonates) from any geographic area. This included randomized controlled trials, prospective cohort studies, retrospective cohort studies, case series, observational epidemiological studies, and brief communications. The following study types were excluded: single case reports; reviews, guidelines, protocols, and modeling studies with no new diagnostic data; *in vitro* laboratory studies that did not diagnose community-acquired infection (e.g., *in vitro* validation studies with spiked samples); studies that tested specimen types other than human blood and did not include human blood (e.g., respiratory tract specimens, stool specimens, and environmental samples). Health care-associated infections and neonatal infections, including stillbirths, were excluded as these syndromes have distinct etiologies from community-onset febrile illness (15). Health care-associated infections were defined as infections that developed during health care or hospitalization and were not present nor incubating at the time of admission (typically at least 48 h after health care/hospital admission) (16).

### Article review

Two reviewers (C.B. and N.G.) independently screened the titles and abstracts of all articles identified through the search to assess their eligibility for full-text review. The same reviewers then evaluated articles deemed potentially relevant to determine final inclusion. Any discrepancies were resolved through discussion, with a third reviewer consulted when necessary.

### Quality assessment for included studies

Two reviewers (C.B. and N.G.) independently evaluated the included articles using a modified Joanna Briggs Institute (JBI) critical appraisal checklist for analytical cross-sectional studies to assess methodological quality and potential bias (Appendix 1: modified JBI checklist for cross-sectional studies) (17). Studies that did not meet the modified JBI criteria were excluded from the analysis.

### Data extraction

Data were manually extracted from the included articles by one author (C.B.) using Microsoft Excel (2019, version 16.72) and independently verified by a second author (N.G.), who identified any discrepancies. Conflicts were resolved with a third reviewer, when necessary. For each included study, we systematically extracted relevant information across several domains. Study-level characteristics encompassed the first author’s last name, year of publication, the country/countries in which the study was conducted, and inclusion of a healthy (non-febrile) control group. Demographic data were collected on participants’ sex, median age, and total sample size. Clinical variables of interest included the presenting syndrome, such as acute febrile illness (AFI) or fever of unknown origin (FUO), as well as 30-day mortality. Pre-analytic variables comprised the extraction method, bead-beating status, and the volumes of blood and DNA used. Diagnostic data captured the number of positive TAC specimens; stratified TAC results by viral, bacterial, parasitic, and fungal etiologies; TAC-determined positivity for more than one pathogen on a single blood specimen; the confirmatory testing method employed; and positive and negative agreement with the confirmatory method, when possible (for organisms identifiable by routine blood culture, we calculated the proportion with discrepant TAC and culture results). All extracted data were pooled for a unified analysis. Reports describing the same data across multiple articles were consolidated into a single record to avoid duplication. Due to possible heterogeneity in primers and probes, data were categorized according to the genus and species whenever possible. *Salmonella enterica* serotypes were grouped according to their association with enteric fever, with typhoidal *Salmonella* referring to *S. enterica* serotypes Typhi and Paratyphi A, B, and C. Culture-negative bacterial pathogens included *Rickettsia* spp., *Coxiella burnetii*, *Bartonella* spp., *Leptospira* spp., *Borrelia* spp., and *Orientia tsutsugamushi*. Culturable organisms refer to bacteria and yeast that standard blood culture methods may identify with a 5-day incubation. *Mycobacterium tuberculosis* complex was reported separately from both culture-positive and culture-negative bacterial infections. Co-infection was defined as two or more organisms in a single blood specimen using TAC.

### Statistical analysis

Descriptive statistics and meta-analyses of proportions using random-effects models were performed using R version 4.2.2 software (2022-10-31). The random-effects model included an inverse variance method with Freeman-Tukey double arcsine transformation, a normal approximation confidence interval (CI) for individual studies, and a continuity correction of 0.5 for studies with zero cell frequencies. We performed the pooled proportion of TAC-detected etiologies of bloodstream infections, mortality rates, co-infection rates, and blood culture positivity (when the latter was used as a confirmatory test). Heterogeneity among eligible articles was assessed using Cochran’s Q statistic (*P*-value < 0.10 for statistical significance) and the *I*^2^ index. The chi-square test was used to analyze categorical variables, such as TAC positivity rates between geographic regions and pathogen detection rates between blood culture and TAC’s ability to detect culturable organisms, with *P*≤ 0.05 considered statistically significant. In the secondary analyses, cases associated with the *Plasmodium* genus were excluded in order to evaluate the incremental diagnostic utility of TAC for identifying the etiology of non-malaria febrile illness. TAC positivity and pathogen identification were also compared across different continents.

## RESULTS

### Search results

**Fig 2:**
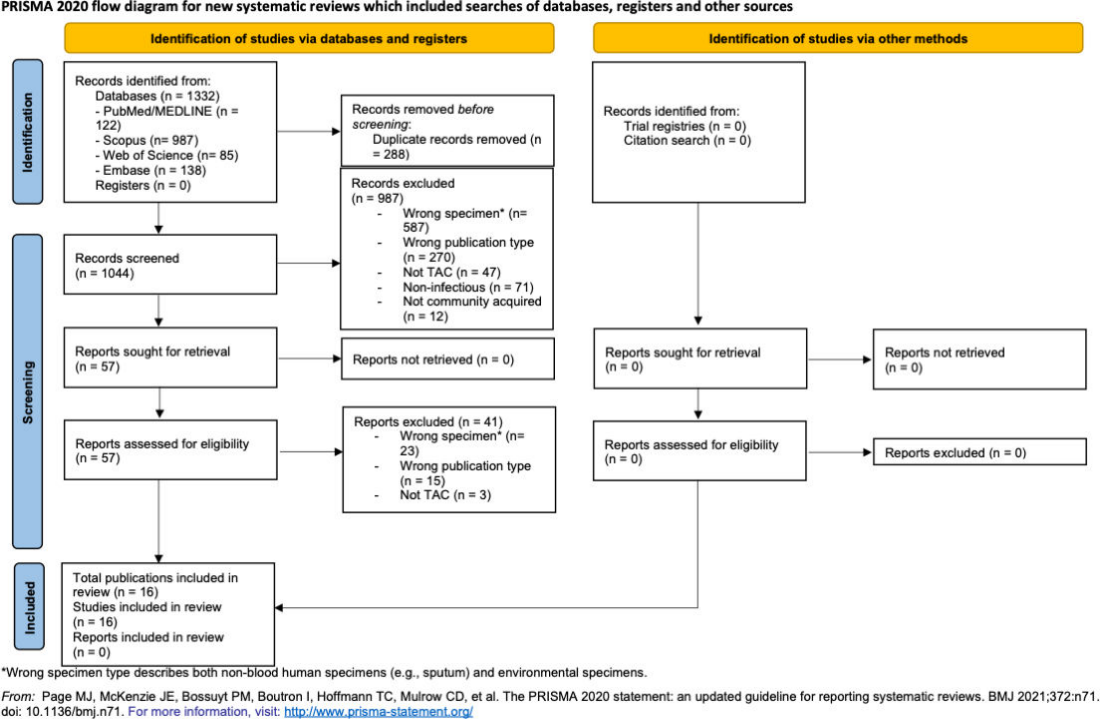
Preferred Reporting Items for Systematic Reviews and Meta-Analyses (PRISMA) 2020 flow diagram.

We identified 1,332 articles through the database search and no supplementary articles through article reference examination (Fig. 2). A total of 288 duplicate articles were removed, leaving 1,044 articles for title and abstract screening. After reviewing titles/abstracts and full texts, 16 publications met the inclusion criteria, describing 8,937 patients with blood specimens tested for infectious pathogens using TAC (Table 1). The included articles were published between 2017 and 2025 and originated from 12 countries, including 10 from Sub-Saharan Africa and two from Asia (Appendix 2: extracted data according to the study and pathogen). No study included a healthy (non-febrile) control group.

**TABLE 1:** Characteristics of studies included in the systematic review^*a*^

Author (year)	Country	Clinical syndrome	Population	N tested	N positive	TAC positivity (%)	Study design	Setting	Case definition/inclusion criteria
Hercik (2017)	Tanzania	AFI	Adults and children	842	421	50.0	Prospective cross-sectional	Hospital	Fever ≥37.5 °C, in prior 5 days
Abade (2018)	Tanzania	AFI outbreak	Adults	141	89	63.1	Outbreak investigation	Outpatient/field	AFI outbreak investigation
Hercik (2018)	Tanzania	FUO	Adults and children	191	60	31.4	Prospective cross-sectional	Hospital	FUO, fever ≥37.5°C
Ali (2020)	Tanzania (Zanzibar)	FUO	Adults	149	27	18.1	Prospective cross-sectional	Hospital	FUO ≥2 weeks, ≥38.3°C
Balish (2021)	Liberia	AFI	Adults	1,506	708	47.0	Prospective cross-sectional	Health centers	Fever ≥37.5°C, in prior 7 days
Marks (2021)	Burkina Faso, Madagascar, and Sudan	AFI	Children and adolescents	615	237	38.5	Retrospective laboratory	Surveillance	Acute fever ≥37.5°C
Rainey (2022)	China (southern)	Acute undifferentiated febrile illness (AUFI)	Adults and children (2–65)	796	341	42.8	Prospective diagnostic	Urban hospital	AUFI ≤7 days, no gastrointestinal (GI)/respiratory symptoms
Ashcroft (2022)	Nigeria	AFI (Lassa-like)	Adults	160	84	52.5	Retrospective laboratory	Laboratory	Febrile illness, Lassa, and malaria testing negative
Das (2022)	Bangladesh	AUFI	Adults and children (≥2 years)	441	27	6.1	Prospective diagnostic	Referral hospital	AUFI ≥38.0°C; no focal signs
Ferdousi (2023)	Bangladesh	AUFI	Adults	120	48	40.0	Prospective cross-sectional	Hospital	AUFI ≥38.4°C
Verani (2024)	Kenya	AUFI	Adults and children (2–65 years)	1,314	599	45.6	Prospective surveillance	Hospital/refugee camp sites	AUFI, ≤2 weeks
Moore (2019)	Uganda	Sepsis (with fever)	Adults	336	336	100	Retrospective laboratory	Referral hospital	Clinical sepsis
Akurut (2025)	Uganda, Kenya, South Sudan, and Democratic Republic of Congo (DRC)	AFI (suspected VHF)	Adults	182	65	35.7	Retrospective cross-sectional	VHF surveillance	Suspected VHF and PCR-negative for VHF
Maro (2025)	Tanzania	AFI	Children (<5 years)	247	50	20.2	Prospective etiologic	Hospital	Axillary temperature ≥38°C
Ngocho (2025)	Tanzania	AFI	Adults and children	697	207	29.7	Prospective cohort	Hospital	Fever ≤72 h or ≥38°C
Courtney et al (2025)	Nigeria	AUFI	Adults and children (≥5 years)	1,200	694	57.8	Prospective diagnostic	Referral hospital	AUFI, ≤10 days

^*a*^ AFI: acute febrile illness, FUO: fever of unknown origin, AUFI: acute undifferentiated febrile illness, VHF: viral hemorrhagic fever, DRC: Democratic Republic of Congo.

### Context of use and case definitions

The intended context of use for TAC on blood varied between diagnostic and surveillance applications. Of the 16 included studies, 10 (63%) evaluated TAC in a prospective diagnostic context, enrolling symptomatic patients with acute or undifferentiated febrile illness to determine the etiology of fever, while 6 (38%) used TAC within retrospective laboratory-based surveillance programs. Undifferentiated and acute febrile illness constituted the primary case definition in 13/16 studies (81%), whereas 3/16 (19%) focused on specific syndromes or surveillance cohorts (e.g., sepsis or viral hemorrhagic fever-suspected cases). Diagnostic studies were predominantly hospital-based (9/10), whereas surveillance studies were mainly conducted within reference laboratory or disease-specific monitoring programs. Fever definitions were heterogeneous, with temperature thresholds ranging from ≥37.5°C to ≥38.4°C and fever duration criteria varying from ≤72 h to over 2 weeks. Exclusion criteria (e.g., absence of focal symptomatology or prior negative testing for malaria or viral hemorrhagic fevers) were inconsistently applied across studies.

### Pre-analytic methodology

Pre-analytic methods were variably reported across the 16 included studies. The nucleic acid extraction methodology was described in 12/16 studies (75%). Of these, 9/12 (75%) used silica-based platforms (High Pure Viral Nucleic Acid Large Volume kits; Roche, Mannheim, Germany), 1/12 (8%) used spin-column methods (QIAGEN, Hilden, Germany), 1/12 (8%) used an automated bead-based MagMAX platform (Thermo Fisher Scientific, Waltham, MA, USA), and 1/12 (8%) used the Quick DNA/RNA Viral kit (Zymo Research, Irvine, CA, USA). Bead-beating was explicitly reported in only 2/16 studies (13%), while 10/16 (63%) reported no bead-beating, and 4/16 (25%) did not report this detail. Blood input volume was reported in 12/16 studies (75%) and varied widely, ranging from 0.5 mL to 5 mL, with most studies using volumes between 2 and 2.5 mL; reported elution volumes ranged from ~40 to 200 µL. Due to substantial heterogeneity and incomplete reporting of pre-analytic variables, formal subgroup or sensitivity analyses assessing their impact on pathogen detection yield were not feasible.

### TAC positivity and demographics

**Fig 3:**
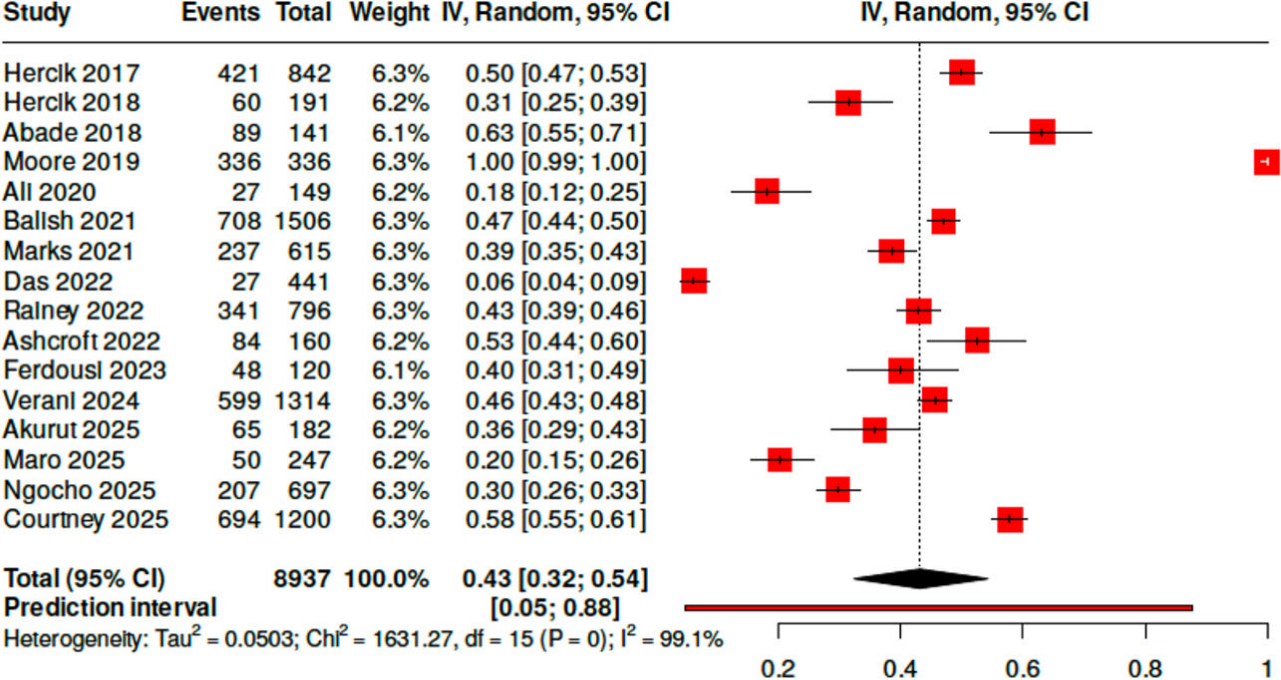
Forest plot of the included studies and their respective percent positivity of TAC performed on blood to identify pathogens associated with community-acquired febrile illness.

TAC detected an organism in the blood of 3,993 of 8,937 participants (0.43, 95% CI [0.32; 0.54]). High heterogeneity existed between the studies (*I*^2^ = 99.1%, Cochran’s Q, *P* < 0.0001, Fig. 3). Parasitic causes of bloodstream infection were most common (0.56, 95% CI [0.54; 0.58]), predominantly due to malaria, with bacterial, viral, and fungal etiologies exhibiting proportions of 0.26 (95% CI [0.25; 0.27]), 0.22 (95% CI [0.20; 0.23]) and 0.005 (95% CI [0.03; 0.09]), respectively. When cases of malaria were excluded, TAC detected an organism in the blood of 1,838 of 8,644 participants (0.21, 95% CI [0.12; 0.45]), again with high heterogeneity (*I*^2^ = 99.0%, Cochran’s Q, *P* < 0.0001). Among all participants, the pooled weighted average age was 25.9 years. Participants had a slight male predominance (0.55, 95% CI [0.50; 0.60]). Of the four studies reporting mortality data for 2,529 participants, pooled 30-day mortality was 0.17 (95% CI [0.10; 0.25]) with significant heterogeneity (*I*^2^ = 95.7%, Cochran’s Q, *P* < 0.0001).

### Parasitic positivity

TAC identified 2,228 cases of febrile illness associated with parasite DNA in blood, of which 2,155 (0.97, 95% CI [0.94; 0.98]) were caused by *Plasmodium* spp., 57 *Schistosoma mansoni* (0.03, 95% CI [0.02; 0.04]), 8 *Leishmania donovani* (0.004, 95% CI [0.002; 0.008]), 7 *Toxoplasma gondii* (0.003, 95% CI [0.001; 0.007]), and 1 case of *Trypanosoma brucei rhodesiense* (0.0004, 95% CI [0.00; 0.003]). Most studies used primers and probes for the *Plasmodium* genus, rather than targets to distinguish between *Plasmodium* species (18–21).

### Bacterial positivity

A total of 1,035 cases of febrile illness with bacterial DNA were identified, associated with 24 bacterial species or species groups from 19 genera (Table 2). There were 375 cases with positivity for culture-positive bacteria (0.36, 95% CI [0.33; 0.39]), 582 cases with positivity for culture-negative bacteria (0.56, 95% CI [0.53; 0.59]), and 78 cases with positivity for *Mycobacterium tuberculosis* complex (0.08, 95% CI [0.06; 0.09]). The most commonly identified bacteria were *Rickettsia* spp., *Streptococcus pneumoniae, Orientia tsutsugamushi, Mycobacterium tuberculosis* complex*,* and *Coxiella burnetii* (Table 2). *Orientia tsutsugamushi* was only identified in Asia.

**TABLE 2:** Frequency and proportion of TAC-detected bacterial pathogens in blood samples from community-acquired febrile illness

Bacterium	Number of cases	Proportion, 95% CI
*Rickettsia* spp.	379	0.37, [0.34; 0.40]
*Streptococcus pneumoniae*	82	0.08, [0.06; 0.10]
*Orientia tsutsugamushi*	81	0.08, [0.06; 0.09]
*Mycobacterium tuberculosis*	78	0.08, [0.06; 0.09]
*Coxiella burnetii*	61	0.06, [0.04; 0.07]
*Brucella* spp.	56	0.05, [0.04; 0.07]
*Escherichia coli*	46	0.04, [0.03; 0.06]
*Leptospira* spp.	39	0.04, [0.03; 0.05]
*Salmonella enterica* typhoidal^*a*^	34	0.03, [0.02; 0.04]
*Salmonella* spp.^*b*^	26	0.02, [0.01; 0.03]
*Enterobacter* spp.	24	0.02, [0.01; 0.03]
*Bartonella* spp.	22	0.02, [0.01; 0.03]
*Pseudomonas aeruginosa*	21	0.02, [0.01; 0.03]
*Klebsiella pneumoniae*	20	0.02, [0.01; 0.03]
*Staphylococcus aureus*	17	0.02, [0.01; 0.02]
*Neisseria meningitidis*	16	0.02, [0.01; 0.02]
*Proteus* spp.	9	0.01, [0.00; 0.01]
*Salmonella enterica* non-typhoidal^*a*^	8	0.01, [0.00; 0.01]
*Acinetobacter baumannii* complex	5	0.00, [0.00; 0.01]
*Aeromonas* spp.	4	0.00, [0.00; 0.01]
*Streptococcus pyogenes*	4	0.00, [0.00; 0.01]
*Klebsiella oxytoca*	1	0.00, [0.00; 0.00]
*Streptococcus suis*	1	0.00, [0.00; 0.00]
*Yersinia pestis*	1	0.00, [0.00; 0.00]

^*a*^ *Salmonella enterica *serotypes were grouped according to their association with enteric fever, with typhoidal *Salmonella *referring to *S. enterica* serotypes Typhi and Paratyphi A, B, and C and non-typhoidal *Salmonella *referring to *S. enterica* serotypes other than Typhi and Paratyphi A, B, and C.

^*b*^ *Salmonella *spp. refers to cases of *Salmonella *bacteremia where the primers in the original studies targeted the *Salmonella *genus with no indication of species or serotype. As cases of *Salmonella* spp. and typhoidal and non-typhoidal *Salmonella enterica* were non-overlapping, cumulatively, there were a total of 68 cases, corresponding to a proportion of 0.07 (95% CI [0.05; 0.08]), attributable to the *Salmonella* genus.

### Viral positivity

Seventeen different viruses were identified by TAC, associated with 870 cases of community-acquired febrile illness (Table 3). When excluding cytomegalovirus (CMV, 174) and Epstein-Barr Virus (EBV, 24), viruses known to reactivate during systemic illness, 672 cases of febrile illness were associated with TAC positivity for a viral pathogen. The most commonly identified viruses were dengue virus, Lassa fever virus, HIV, chikungunya virus, Rift Valley fever virus, and O’nyong’nyong virus (Table 3). Dengue (DENV 1-4) was a common viral etiology in both African and Asian continents, though it accounted for a disproportionately higher proportion of viral positivity in Asia than Africa (0.99, 95% CI [0.97; 1.00] vs 0.21, 95% CI [0.18; 0.25]; *P* < 0.001). Although Lassa fever virus was most frequently identified among specimens from Africa, this finding was largely driven by the study by Courtney et al. in Nigeria; in the remaining studies, dengue virus was the most commonly detected viral pathogen (21).

**TABLE 3:** Frequency and proportion of TAC-detected viral pathogens in blood samples from community-acquired febrile illness

Virus	Number of cases	Proportion, 95% CI
Dengue virus^*a*^	350	0.40 [0.37; 0.43]
Lassa fever virus	187	0.21 [0.19; 0.24]
Cytomegalovirus^*b*^	174	0.20 [0.17; 0.23]
HIV	51	0.06 [0.04; 0.07]
Chikungunya virus	36	0.04 [0.03; 0.05]
Epstein–Barr virus^*b*^	24	0.03 [0.02; 0.04]
Rift Valley fever virus	12	0.01 [0.01; 0.02]
O'nyong'nyong virus	7	0.01 [0.00; 0.01]
Hepatitis E	6	0.01 [0.00; 0.01]
Yellow fever virus	5	0.01 [0.00; 0.01]
Crimean-Congo hemorrhagic fever virus	5	0.01 [0.00; 0.01]
West Nile virus	3	0.00 [0.00; 0.01]
Mpox virus (Orthopoxvirus)^*c*^	3	0.00 [0.00; 0.01]
Adenovirus	2	0.00 [0.00; 0.01]
Enterovirus	2	0.00 [0.00; 0.01]
Zika virus	2	0.00 [0.00; 0.01]
Measles virus	1	0.00 [0.00; 0.00]

^*a*^ Dengue virus includes all four dengue virus serotypes, DENV-1, DENV-2, DENV-3, and DENV-4. HIV: human immunodeficiency virus.

^*b*^ Certain viruses, such as EBV and CMV, likely reflect reactivation and are unlikely to be responsible for the presenting febrile illness.

^*c*^ As Orthopoxvirus and Mpox virus detections were non-overlapping, these were combined; cumulatively, there were a total of three cases attributable to Orthopoxviruses.

### Fungal positivity

Only 21 cases of fever were associated with fungal DNA in the blood. There were 11 cases of *Candida* spp., 9 cases of *Cryptococcus neoformans,* and 1 case of *Histoplasma capsulatum*.

### Co-infection rates

**Fig 4:**
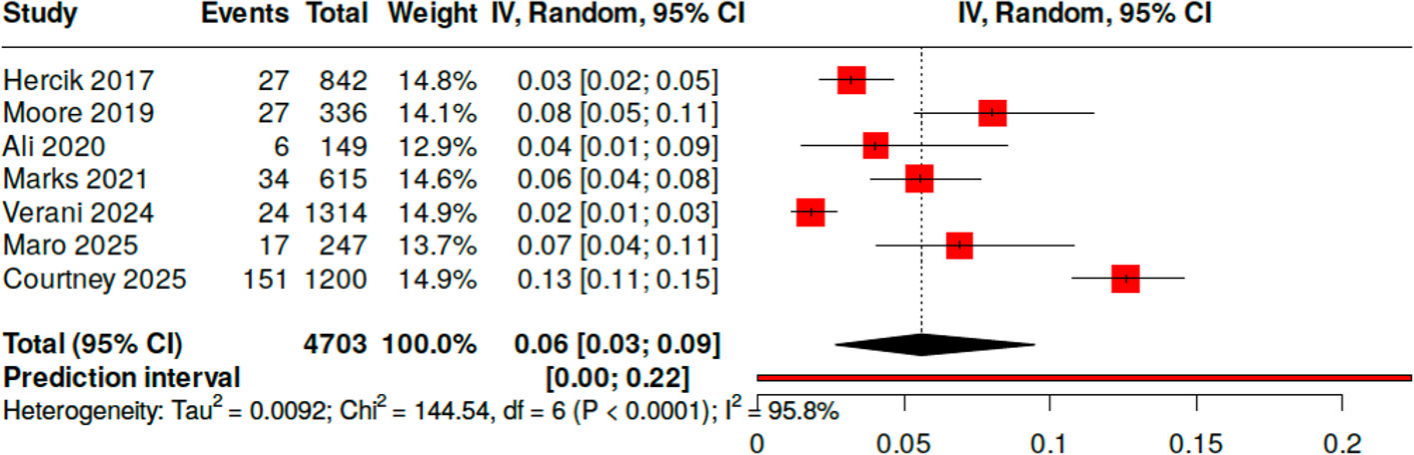
Forest plot of co-infection rates identified by TAC on blood.

A total of 7 studies, analyzing blood specimens from 4,703 participants, described cases where more than one organism was identified in a single TAC result (2, 19–25). The pooled co-infection rate was 0.06 (95% CI [0.03; 0.09]), with substantial heterogeneity across studies (*I²* = 95.8%, Cochran’s Q = 144.5, *P* < 0.001) (Fig. 4). When excluding CMV and EBV, the most commonly identified co-infections involved *Plasmodium* spp. with either *Rickettsia* spp.*,* HIV, dengue, or *Salmonella* spp. (2, 20–22, 25). Documented bacterial co-infection with *Plasmodium* spp. or dengue virus included *Salmonella* spp., *Brucella* spp., *Staphylococcus aureus, Rickettsia* spp. or other culture-negative bacteria (20, 22). A total of 87 cases of polymicrobial results involved *Plasmodium* spp. and culture-negative bacteria, including 85 cases with *Rickettsia* spp. and 2 with *Bartonella* spp. (20–23, 25). A total of 25 individuals had positive TAC results for both *Rickettsia* spp. and Lassa fever virus, and 9 individuals had positive results for both *Rickettsia* spp. and *Brucella* spp. (21, 25) A total of 4 individuals had positive TAC results for both dengue virus and *Brucella* spp., 1 individual had a positive TAC result for dengue virus and *Rickettsia* spp., and 1 had a positive result for *Brucella* spp. and *S. aureus* (23).

### Comparative microbiologic testing

Thirteen studies included at least one additional diagnostic modality other than TAC. Nine studies included blood culture and two included sequencing (2, 26). Two studies used targeted qPCR, two used Giemsa-stained blood films for malaria diagnosis, and three employed malaria rapid diagnostic tests (mRDT) detecting HRP2 and pLDH antigens (2, 26–30). Agreement between blood culture and TAC was high, with a positive percent agreement of 0.73 (95% CI [0.53; 0.89]) and a negative percent agreement of 0.98 (95% CI [0.95; 1.00]) (Table 4). Among 2,638 patients with available data, pooled blood culture positivity was 0.06 (95% CI [0.01; 0.16]) and demonstrated substantial heterogeneity across studies (*I²* = 98.0%, Cochran’s Q, *P* < 0.01). This was significantly lower than the pooled TAC positivity for culturable bacteria and fungi (0.14, 95% CI [0.04; 0.20], *P* < 0.001). Consistent with this, the proportion of TAC-positive/culture-negative results exceeded that of TAC-negative/culture-positive results (0.09 vs 0.02, *P* < 0.001).

**TABLE 4:** Diagnostic agreement and detection yield of blood culture versus blood TAC for culturable organisms^*a*^

Metric	Estimate	95% CI	Interpretation
Positive percent agreement (PPA)	0.73	[0.53; 0.89]	Concordance when organisms were detected by both methods
Negative percent agreement (NPA)	0.98	[0.95; 1.00]	Concordance for negative results
Blood culture positivity proportion	0.06	[0.01; 0.16]	Pooled estimate across studies
TAC positivity proportion (culturable bacteria/fungi)	0.14	[0.04; 0.20]	Pooled estimate across studies. Higher than blood culture (*P* < 0.001)
TAC-positive, culture-negative	0.09	[0.02; 0.22]	Additional detections by TAC
TAC-negative, culture-positive	0.02	[0.01; 0.05]	Detections missed by TAC

^*a*^ TAC, TaqMan Array Card. Pooled estimates were derived using random-effects meta-analysis where applicable. Blood culture positivity was assessed among 2,638 patients and demonstrated substantial heterogeneity (I^2^ = 98.0%, Cochran’s Q, *P* < 0.01). Positive and negative percent agreement were used in lieu of sensitivity and specificity because of the lack of a perfect reference standard. Culturable organisms refer to bacteria and yeast that standard blood culture methods may identify with a 5-day incubation.

Three studies compared mRDTs with TAC, the latter detecting *Plasmodium* at the genus level (24, 27, 30). In a high-transmission setting in Tanzania, Hercik et al. reported malaria positivity in 34% of patients by mRDT, compared with 47% by TAC (24). Ngocho et al. demonstrated high concordance between mRDT and TAC (positive percent agreement [87.5%] and negative percent agreement [96.7%]), although TAC identified additional *Plasmodium* infections not detected by mRDT (30). In contrast, Rainey et al. observed complete discordance in a low-transmission setting as none of 10 mRDT-positive cases were confirmed by TAC, suggesting possible false-positive mRDT results (27).

### Geographic variation in pathogen identification

TAC positivity was higher in African than in Asian studies (0.47, 95% CI [0.46; 0.48] vs 0.31, 95% CI [0.28; 0.33], *P* < 0.01), though substantial heterogeneity existed for both continents (*I*^2^ = 99.0%, Cochran’s Q, *P* < 0.01 for both African and Asian studies). Each continent exhibited distinct patterns of pathogen identification. In African studies, positivity was dominated by *Plasmodium* spp., which accounted for 64.4% (2,251/3,493) of all positive results, whereas in Asia, it was responsible for only 2.4% (10/416) of TAC positivity. Excluding *Plasmodium* spp. and CMV, the most commonly identified organisms in African studies were *Rickettsia* spp. (379/3,493, 10.9%), Lassa fever virus (187/3,493, 5.4%), dengue virus (140/3,493, 4.0%), *Streptococcus pneumoniae* (82/3,493, 2.3%), *Mycobacterium tuberculosis* complex (78/3,493, 2.2%), and *Salmonella* spp. (68/3,493, 1.9%). In contrast, among Asian studies, the predominant organisms were dengue virus (210/416, 50.5%), *Orientia tsutsugamushi* (81/416, 19.5%), *Coxiella burnetii* (42/416, 10.1%), *Rickettsia* spp. (19/416, 4.6%), and *Salmonella* spp. (18/416, 4.3%).

### Quality assessment

Quality assessment using the JBI critical appraisal checklist revealed that 34.8% of publications had sufficient methodological detail and outcome data to be included in the full-text analysis (Appendix 3: modified JBI quality assessment and rationale). The majority of excluded studies were removed due to use of non-blood specimens, *in vitro* or validation-only designs, non–TAC-based diagnostics, or pathogen-specific analyses without representative bloodstream infection data (Appendix 3) (17).

## DISCUSSION

This systematic review of TAC-based studies on community-acquired febrile illness identifies *Plasmodium* spp., dengue virus, and *Rickettsia* spp. as the most frequently detected pathogens. These findings partially overlap with prior systematic reviews that have consistently highlighted malaria and dengue as predominant causes of febrile illness—particularly in Sub-Saharan Africa and Southeast Asia, respectively—while *Rickettsia* spp. have been less prominently reported (31, 32). The broad analyte coverage of TAC uncovered additional clinically significant pathogens, including *Orientia tsutsugamushi*, *Coxiella burnetii*, and *Mycobacterium tuberculosis* complex, as well as viral pathogens such as Lassa fever virus, O’nyong’nyong virus, and Rift Valley fever virus in African studies (2, 27, 30). This list differs from previous systematic reviews on bloodstream infections that predate TAC and identify *Salmonella enterica* as the most common non-malarial cause of bloodstream infection (31). While further studies are required, TAC’s expanded pathogen detection highlights the value of high-throughput molecular platforms for etiologic characterization.

TAC positivity was significantly higher among African studies compared to Asian studies, a difference that appeared largely driven by the predominance of *Plasmodium* spp. in African cohorts. This finding likely reflects the well-established geographic distribution of malaria. The overrepresentation of *Plasmodium* spp. in African TAC results may therefore inflate overall positivity estimates relative to Asia, where arboviral and rickettsial pathogens were more prominent. Nevertheless, even when cases with *Plasmodium* positivity were excluded, TAC detected a pathogen in 21.0% of participants, underscoring its diagnostic utility for identifying alternative etiologies of febrile illness in both malaria-endemic and non-endemic settings.

Across included studies, TAC was applied in two distinct contexts: prospective diagnostic evaluation and retrospective surveillance. In diagnostic settings, TAC was used as a multiplex tool to characterize the etiologic spectrum of acute or undifferentiated febrile illness, often without a uniform reference standard. In contrast, surveillance applications analyzed archived specimens within disease-specific programs (e.g., sepsis, typhoid fever, or viral hemorrhagic fever), with inclusion criteria commonly defined by prior negative testing or programmatic case definitions rather than clinical decision-making. These differing contexts of use explain observed heterogeneity in enrollment criteria and limit direct comparison of diagnostic performance metrics across studies.

In many Sub-Saharan African settings where TAC studies were conducted, diagnostic algorithms for febrile illness often rely on syndromic assessment in the context of limited diagnostic capacity, increasing the risk of etiologic misclassification (1). Patients with bacterial bloodstream infections may be empirically treated for malaria, especially in endemic regions where malaria remains a presumptive diagnosis for fever (1, 33). One article in this systematic review analyzed a subgroup of 17 participants with a clinical malaria diagnosis in rural Tanzania (23). Within this group, TAC detected 2 cases of dengue, 2 cases of rickettsiosis, 1 of brucellosis, 1 of Q fever, and 1 of *Streptococcus pyogenes* (23). The importance of molecular diagnostics to correctly identify pathogens in the bloodstream is particularly relevant to pathogens commonly identified using nonspecific serologic assays; in a study of febrile illness etiologies using 16S rRNA metagenomic sequencing on blood specimens, cases of *Coxiella burnetii* and *Bartonella quintana* infection were initially misdiagnosed as leptospirosis due to false-positive microscopic agglutination testing (34).

A strength of TAC is its ability to detect intracellular vector-borne and zoonotic bacteria that cannot be identified using routine culture. Over half of cases with bacterial DNA identified in this review were due to culture-negative bacteria such as *Rickettsia* spp.*, Orientia tsutsugamushi*, *Coxiella burnetii*, *Leptospira* spp., and *Bartonella* spp. (2, 27, 30). These pathogens are underrepresented in culture-based studies and are largely absent from global prioritization frameworks (35, 36). Their detection in diverse settings, including high incidence reports from Nigeria, South India, Madagascar, and Tanzania, suggests that these pathogens may be more common than currently appreciated (2, 10, 12, 21, 23). This phenomenon has been reported from studies using multiplex qPCR platforms other than TAC. In an Ethiopian study of febrile illness etiologies, *Borrelia* and *Rickettsia* spp. were more commonly identified than *Plasmodium* spp. (37). Culture-negative bacteria have also been identified among febrile travelers returning to Europe with non-malarial illness, many of whom were diagnosed with infections caused by *Rickettsia* spp., *Coxiella burnetii*, *Bartonella* spp., *Leptospira* spp., and *Anaplasma phagocytophilum* (38). Incorporating such pathogens into diagnostic algorithms, surveillance programs, and guideline development is essential to improve public health planning, especially in areas where the burden of vector-borne and zoonotic diseases is greater.

Co-infections were identified in 6% of patients tested, with *Plasmodium* spp. most frequently involved in polymicrobial results. Common pairings included malaria with dengue virus, HIV, or bacterial pathogens such as *Rickettsia* spp., *Salmonella* spp., *Brucella* spp., and *Bartonella* spp. (2, 20, 22, 23, 25, 39). While the concurrence of *P. falciparum* malaria and nontyphoidal *Salmonella* is well described among children in Sub-Saharan Africa, further studies are required to elucidate the clinical significance of polymicrobial infections with other bacterial species (25, 40, 41).

Most studies employed at least 1 additional diagnostic modality, most commonly blood culture. Agreement between TAC and blood culture was moderate for positive results (73%) but high for negative results (98%), indicating strong concordance in ruling out infection.

These numbers are similar to reports from *in vitro* TAC validation studies using clinical and spiked specimens (7). The pooled proportion of pathogen detection by standard blood culture was low (6%), consistent with other reports from Sub-Saharan Africa (42). In contrast, TAC identified culturable pathogens in a higher proportion of cases (14%). Additional studies are necessary to interrogate this discrepancy further as culture is often viewed as the gold-standard test. Although Abade et al. suggested that TAC may be less costly than performing a broad range of conventional microbiologic assays for diverse pathogens, the platform’s high capital investment (approximately USD 100,000), per-specimen reagent costs (USD 50–100), requirement for a separate nucleic acid extraction step, and incompatibility with many thermocycler models may limit its feasibility (27). Beyond financial barriers, TAC does not provide phenotypic antimicrobial susceptibility testing, a critical component of bacteriologic diagnostics. Moreover, even in well-resourced laboratory settings, turnaround times are likely to exceed 24 h, which may constrain the assay’s usefulness for acute clinical decision-making.

This review has several limitations. It is limited by few included studies, which exhibited substantial heterogeneity in design, geographic representation, and content, limiting direct comparability. All studies were conducted in Sub-Saharan Africa or two countries in Asia, limiting generalizability outside these areas. TAC’s performance depends on the selected primer-probe sets, which may vary across studies and further restrict generalizability. TAC positivity rates must be interpreted cautiously as one sepsis study included CMV and EBV viral reactivation in their positivity rates, without sufficient evidence for clinical disease (19). More broadly, PCR does not in itself establish causality with clinical disease as nucleic acid detection cannot distinguish between viable and nonviable organisms nor between asymptomatic carriage, latent infection, or active disease. Thus, there remains a substantial risk of overinterpretation and over-attribution of TAC results without clinical contextualization. In the ANISA trial, which used TAC to investigate the etiologies of neonatal illness, blood-based TAC detection of *Neisseria gonorrhoeae*, *Salmonella* spp., and *Escherichia coli* did not differ between symptomatic infants and healthy controls (43). A similar pattern has been observed for PCR detection of culture-negative bacterial pathogens. A study conducted in Malaysian Borneo reported frequent *Leptospira* spp. PCR positivity in blood specimens from asymptomatic sanitation workers (44). In addition, findings from the SAFIAN study in Nigeria showed that patients with *Rickettsia* or *Plasmodium* spp. coinfections were not clinically distinguishable from those with single-pathogen infections in terms of illness severity (25). Among articles included in our systematic review, none incorporated a healthy control group, and confirmatory testing was inconsistently applied. TAC’s diagnostic accuracy, though promising, may require additional validation in various settings. The data may be influenced by bias present in the original studies, including publication bias. Furthermore, clinical data were insufficient to assess TAC’s impact on clinically relevant outcomes as TAC may identify subclinical infections. These issues underscore the importance of well-designed case-control studies to accurately assess the clinical relevance of blood-based TAC results across diverse etiologies, syndromes, and epidemiological settings.

In summary, blood-based TAC enables broad, culture-independent pathogen detection in community-acquired febrile illness and consistently identifies *Plasmodium* spp., dengue virus, *Rickettsia* spp., and other culture-negative bacterial pathogens. TAC also detects polymicrobial infections that are frequently missed by routine diagnostics and surveillance programs. Across studies, TAC was applied in two contexts—prospective diagnostic evaluation of febrile illness and retrospective surveillance of archived specimens. While TAC may support etiologic diagnosis of febrile illness in individual patients, results require cautious interpretation to distinguish nucleic acid positivity from active disease. Its multiplex capacity and species-level resolution make it well suited for epidemiologic surveillance.

## SUPPLEMENTAL MATERIAL

File S1 jcm.00101-26-s0001.docx Modified JBI checklist.

File S2 jcm.00101-26-s0002.xlsx Extracted data according to study and pathogen.

File S3 jcm.00101-26-s0003.xlsx Modified JBI quality assessment and rationale.

ASM does not own the copyrights to Supplemental Material that may be linked to, or accessed through, an article. The authors have granted ASM a non-exclusive, world-wide license to publish the Supplemental Material files. Please contact the corresponding author directly for reuse.
